# Inhibition of *miR-143* during ischemia cerebral injury protects neurones through recovery of the hexokinase 2-mediated glucose uptake

**DOI:** 10.1042/BSR20170216

**Published:** 2017-07-04

**Authors:** Xianzhu Zeng, Na Liu, Jing Zhang, Lei Wang, Zhecheng Zhang, Ju Zhu, Qian Li, Yuwen Wang

**Affiliations:** 1 Department of Neurology, Third Central Hospital of Tianjin, Tianjin 300170, China; 2 Department of Neurology, Tianjin Institute of Hepatobiliary Disease, Tianjin 300170, China; 3 Department of Neurology, Tianjin Key Laboratory of Artificial Cell, Tianjin 300170, China; 4 Department of Neurology, Artificial Cell Engineering Technology Research Center of Public Health Ministry, Tianjin 300170, China; 5 Department of Neurology, The Third Central Clinical College of Tianjin Medical University, Tianjin 300170, China

**Keywords:** Hexokinase 2, ischemia cerebral injury, miR-143

## Abstract

Ischemic stroke, a major cause of death, is caused by occlusion of a blood vessel, resulting in significant reduction in regional cerebral blood flow. MiRNAs are a family of short noncoding RNAs (18–22 nts) and bind the 3′-UTR of their target genes to suppress the gene expression post-transcriptionally. In the present study, we report that *miR-143* is down-regulated in rat neurones but highly expressed in astrocytes. *In vivo* middle cerebral artery occlusion (MCAO) and *ex vivo* oxygen-glucose deprivation (OGD) results showed that miR-143 was significantly induced by ischemia injury. Meanwhile, we observed suppression of glucose uptake and lactate product of rat brain and primary neurones after MCAO or OGD. The glycolysis enzymes hexokinase 2 (HK2), PKM2, and LDHA were inhibited by MCAO or OGD at protein and mRNA levels. In addition, overexpression of miR-143 significantly inhibited HK2 expression, glucose uptake, and lactate product. We report that HK2 is a direct target of miR-143. Importantly, restoration of HK2 in miR-143 overexpressing rat neurones recovered glucose uptake and lactate product. Our results demonstrated inhibition of miR-143 during OGD could protect rat neuronal cells from ischemic brain injury (IBI). In summary, the present study reveals a miRNA-mediated neuron protection during IBI, providing a new strategy for the development of therapeutic agents against IBI.

## Introduction

Brain ischemic stroke, which is a major cause of death, ranks number 3 amongst all the causes of death, after cardiovascular disease and cancer [1]. Ischemic stroke is caused by occlusion of a blood vessel, resulting in significant reduction in regional cerebral blood flow [2]. Consequently, brain damage arises from deprivation of oxygen and glucose due to blockage of local blood supply. Cerebral ischemia activates a series of signaling cascades which lead to neurones’ death [3]. It has been known that oxidative stress induced by cerebral ischemia is one of the major causes of the neuronal injury [4]. However, the pathophysiological mechanisms are not still under investigation. Therefore, revealing the molecular mechanism for ischemic injury is necessary to develop effective therapies.

MiRNAs are a family of short noncoding RNAs (18–22 nts) and bind the 3′-UTR of their target genes as a post-transcriptional suppression in various cell types [5]. Recent review articles summarized the roles and mechanisms of miRNA-induced regulation of cellular and molecular processes in response to hypoxic and/or ischemic stress [6,7]. They described expressions of various groups of miRNAs that were transcriptionally changed during the process of brain ischemia injury [6,7]. Moreover, a study reported that miR-210 is induced by ischemic brain injury (IBI) and inhibition of miR-210 provided neuroprotection in neonatal rats [8], suggesting a potentially therapeutic role of miRNAs in IBI.

Continuous energy supply is important for maintenance of brain functions by oxidative metabolism of oxygen and glucose [9]. Glucose is the major nutrition source used by the brain under normal conditions [9]. Therefore, inadequate supply of oxygen or glucose could result in cognitive dysfunction, neuronal cell death, and persistent brain damage [10]. Currently, the roles of miRNAs in the cellular metabolism and ischemia injury of brain are unclear. In the present study, we will investigate the function of miR-143 in the regulation of glucose metabolism. The correlation of miR-143 and IBI will be assessed.

## Materials and methods

### Cell culture

Primary rat brain cortex neurones were purchased from Lonza (Basel, Switzerland) and cultured in primary neuron growth medium (PNGM) (Lonza, Basel, Switzerland) with 10% FBS (Invitrogen) plus 1× penicillin/streptomycin. Primary rat microglia cells were purchased from Lonza (Basel, Switzerland) and cultured in DMEM (high glucose) with 10% FBS (Invitrogen) plus 1× penicillin/streptomycin. Primary rat cortical astrocytes were purchased from Thermofisher Scientific (Waltham, MA) and cultured in DMEM (high glucose) with 10% FBS (Invitrogen) plus 1× penicillin/streptomycin. All cells were cultured at 37°C with 5% CO_2_. The cultured neurones under the oxygen-glucose deprivation (OGD) conditions *in vitro* were transferred into an anaerobic chamber containing a gas mixture composed of 5% CO_2_ and 95% N_2_. The culture medium was replaced with deoxygenated, glucose-free Hanks’ Balanced Salt Solution (Invitrogen, Carlsbad, CA) and cells were maintained in the hypoxic chamber for 24 h, then, returned to regular 37°C, 5% CO_2_ and glucose-containing medium. Control cells received no treatment.

### Rat ischemia stroke model

The rat ischemia stroke model was established according to a previous report [11]. Adult male Wistar rats (weight: 246 ± 10 g) were purchased from the Chinese Academy of Sciences (Shanghai, China). Rats were bred and maintained in a pathogen-free facility and were subjected to middle cerebral artery occlusion (MCAO) as previously described [11]. After anesthesia by sodium pentobarbital (100 mg/kg), the right common carotid artery, external carotid artery, and internal carotid artery of rats were exposed through a midline cervical incision. A piece of monofilament nylon suture was inserted through the right internal carotid artery to the base of the middle cerebral artery, resulting in preventing blood flow to the cortex and striatum for 90 min. The rats were then recovered for 1 day. The total brains were then removed for downstream assays of the present study. The sham control rats were subjected to similar surgeries to expose the carotid arteries but without occlusion of the middle cerebral artery. The present study was approved by the Animal Care Committee at the Third Central Hospital of Tianjin. Animals were operated in accordance with the institutional guidelines for animal care.

### MiRNA transfection

The control miRNA mimic, miR-143 mimic, miR-143 inhibitor, or negative control was transfected at a concentration of 200 nM using Lipofectamine 2000 (Invitrogen, Carlsbad, California, U.S.A.) according to the manufacturer’s protocol. After 48 h, cells were collected and subjected to the downstream assays.

### qRT-PCR

Total miRNA from the cultured cells was extracted using the miRNeasy Mini Kit (Qiagen, Hilden, Germany), according to the manufacturer’s instructions. The concentration and purity of RNA extracts were measured by NanoDrop Spectrophotometer2000 (ThermoFisher, Waltham, Massachusetts, U.S.A.). For detecting miR-143 expressions, cDNA was synthesized using the Mir-X™ miRNA First-Strand Synthesis Kit (Takara, Dalian, Liaoning, China). The following thermocycles was used: 37°C for 60 min and 85°C for 5 min. Real-time quantitative PCR was performed using SYBR Premix Ex Taq™ II (Takara, Dalian, Liaoning, China) with the following amplification conditions: 95°C for 10 s, followed by 40 cycles of 95°C for 5 s, 62°C for 20 s, and 72°C for 30 s. The reactions were analyzed by the Light Cycler s480 Real Time PCR System (Roche, Mannheim, Baden-Wüerttemberg, Germany). U6 expression was used as the internal control for detecting miR-143 expressions. The measurements of glycolysis mRNAs were performed according to the previous report [12]. The relative expression levels of miRNA and mRNA were quantitated using the 2^−ΔΔ*C*^_t_ method.

### MiRNA target prediction

The prediction and analysis of potential targets of miR-143 were performed using the miRNA target prediction software program: TargetScan (http://www.targetscan.org/).

### Luciferase assays

A total of 1 × 10^5^ 293T cells per well were seeded in a 24-well plate overnight. Cells were then cotransfected with 50 nM miR-143 mimic or control mimic, and 200 ng wild-type or mutant hexokinase 2 (HK2) 3′-UTR vectors using Lipofectamine 2000 (Invitrogen, Carlsbad, CA, U.S.A.), according to the manufacturer’s instructions. After 48 h, the 293T cells were collected, and the luciferase activity was measured using the Dual-Glo luciferase assay system (Promega, Madison, Wisconsin, U.S.A.) according to the manufacturer’s instructions. Firefly luciferase activity was used as an internal control. Experiments were performed in triplicate.

### Cell survival

Neurones’ survival rates were assessed by the MTT assay. Cells were plated in 96-well plates and grown overnight until 70–80% confluence. After treatment with OGD, MTT was added to each well under sterile conditions and the plates were incubated for 4 h at 37°C. Untransformed MTT was removed by aspiration, and formazan crystals were dissolved in DMSO (150 μl/well). Results were quantitated spectroscopically at 540 nm using Bio–Rad Automated EIA Analyzer (Bio–Rad). The experiments were performed in triplicate.

### Caspase-3 activity assay

Caspase-3 activity assays were performed using Caspase-Glo® 3/7 Assay kit (Promega, Madison, WI, U.S.A.) according to the manufacturer’s protocol. Luminescence was measured using a microplate reader Victor X3 from PerkinElmer (Waltham, MA).

### Glucose uptake and lactate product assays

The glucose uptake and lactate product assays were performed using the Glucose Uptake Colorimetric Assay Kit and Lactate Colorimetric Assay Kit (BioVision, Milpitas, California, U.S.A.) according to the manufacturer’s protocol. Experiments were performed in triplicate.

### Western blot

The whole cell proteins were extracted using RIPA lysis buffer (Beyotime, Haimen, Hainan, China). The protein concentrations were measured by the Bradford assay. Equal amount s of each sample of proteins were denatured at 99°C for 5 min in loading buffer. Then, samples were electrophoresed on SDS/PAGE (10% gels), then transferred on to PVDF membranes (Miliipore Corp, Billerica, Massachusetts, U.S.A.). The membranes were washed and blocked with 4% nonfat milk for 1 h at room temperature. Membranes were incubated with primary antibodies against HK2 (1:1000, #2867, Cell Signaling), PKM2 (1:1000, #4053, Cell Signaling), LDHA (1:1000, #3582, Cell Signaling) and GAPDH (1:4000, #5174, Cell Signaling) overnight at 4°C. Membranes were washed and incubated with an anti-rabbit secondary antibody for 2 h at room temperature. GADPH was used as an internal control. The protein bands were imaged using a LI-COR Odyssey imaging system (LI-COR Bioscience, Lincoln, Nebraska, U.S.A.).

### Statistical analysis

All experiments were performed in triplicate. Results were shown as mean ± S.E.M. Statistical difference was measured by a two-tailed unpaired Student’s *t* test (Prism 5.0). *P*<0.05 is considered significant.

## Results

### Expression of miR-143 is low in rat neurones

**Figure 1:**
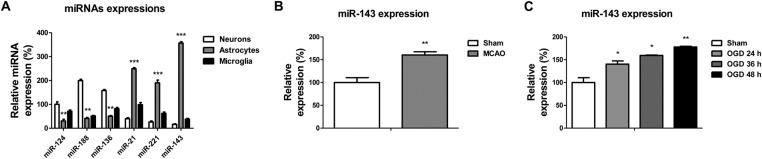
MiR-143 is down-regulated in rat neurones and induced by IBI (**A**) Expressions of miRNAs in rat neurones, astrocytes, and microglia. (**B**) Expression of miR-143 was detected by qRT-PCR without or with transient MCAO. (**C**) Expression of miR-143 in rat neurones was detected by qRT-PCR without or with oxygen and glucose deprivation at 24, 36, and 48 h. Data are representative of three independent experiments. Mean ± S.E.M. *, *P*<0.05; **, *P*<0.01; ***, *P*<0.001.

To address the question of whether miR-143 expression in brain has cell-type specificity, we first evaluated the expression levels of miR-143 and other miRNAs in three distinct neural cell types: neurones, astrocytes, and microglia. Interestingly, we observed that miR-124, miR188, and miR-136 were specifically enriched in neurones but down-regulated in astrocytes (Figure 1A). In addition, miR-143 was significantly down-regulated in neurones and microglia, but higher expression of miR-143 was observed in astrocytes (Figure 1A), suggesting that miR-143 might involve in the maintenance of neuron functions during IBI.

### MiR-143 is up-regulated in rat IBI

To investigate the role of miR-143 in regulating IBI, we compared the expression levels of miR-143 in rat brain before and after transient MCAO, which presents an efficient model of inducing reproducible transient or permanent ischemia of the middle cerebral artery. Our results demonstrated that miR-143 expression was significantly induced in the rat brain after MCAO compared with the sham control (Figure 1B). To strengthen the above *in vivo* results, we established an *ex vivo* stroke model using the primary rat neuron cells with OGD, which is a widely used model for stroke, and shows similarities with the *in vivo* models of brain ischemia [11]. Consistently, the expression of miR-143 was significantly induced by the OGD for 24, 36, and 48 h (Figure 1C). Taken together, our results suggested a regulatory role of miR-143 during IBI in rat neurones.

### Glucose uptake and lactate product is compromised by IBI

**Figure 2:**
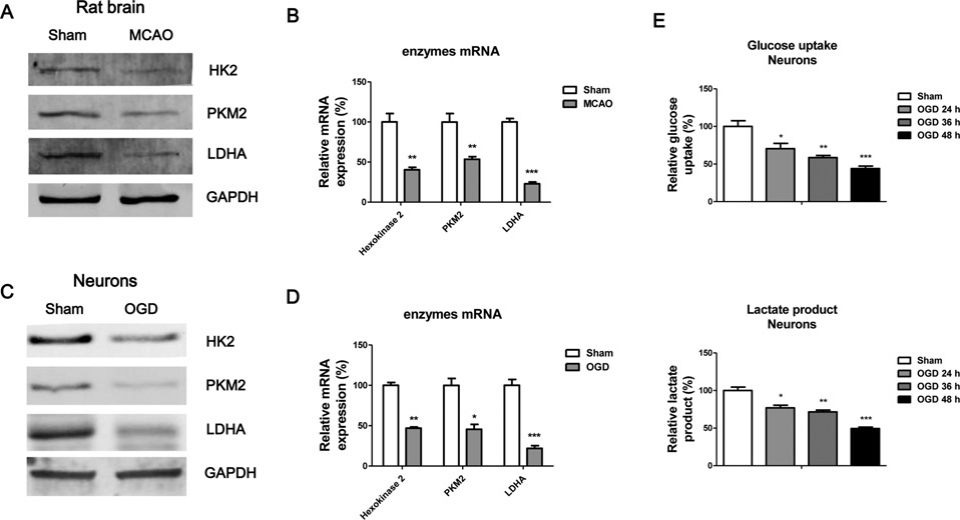
Glucose uptake and lactate product are impaired under IBI Rats were subjected to MCAO, followed by (**A**) Western blot analysis and (**B**) measurements of mRNA expressions of glycolysis key enzymes of the rat brains. GAPDH was the loading control. (**C**) The expressions of glycolysis key enzymes were measured by Western blot and (**D**) qRT-PCR in rat neurones without or with OGD. (**E**) Glucose uptake (upper) and lactate product (lower) were measured in rat neurones without or with OGD at 24, 36, and 48 h. Data are representative of three independent experiments. Mean ± S.E.M. *, *P*<0.05; **, *P*<0.01; ***, *P*<0.001.

It is well studied that brain has a continuous demand of energy met by glucose and oxygen metabolism [13]. Importantly, the nutrition supply is compromised in the injured brain and inadequate supply will exacerbate tissue damage [14]. We compared the glucose metabolism key enzymes’ expressions in rat brains during MCAO, results in Figure 2A,B demonstrated the protein and mRNA expressions of HK2, PKM2 and LDHA were significantly down-regulated by IBI. Consistently, the protein and mRNA expressions of HK2, PKM2, and LDHA were significantly down-regulated in neurones during OGD (Figure 2C,D). To assess whether glucose uptake and lactate product were dysregulated during neuron OGD, we compared the glucose uptake and lactate product rates of rat primary neurones without or with OGD at 24, 36, or 48 h. As we expected, results demonstrated decreased glucose uptake and lactate product during neurones OGD (Figure 2E), suggesting restoration of glucose metabolism might contribute to diminish the IBI.

### MiR-143 suppresses glucose uptake through targetting HK2

**Figure 3:**
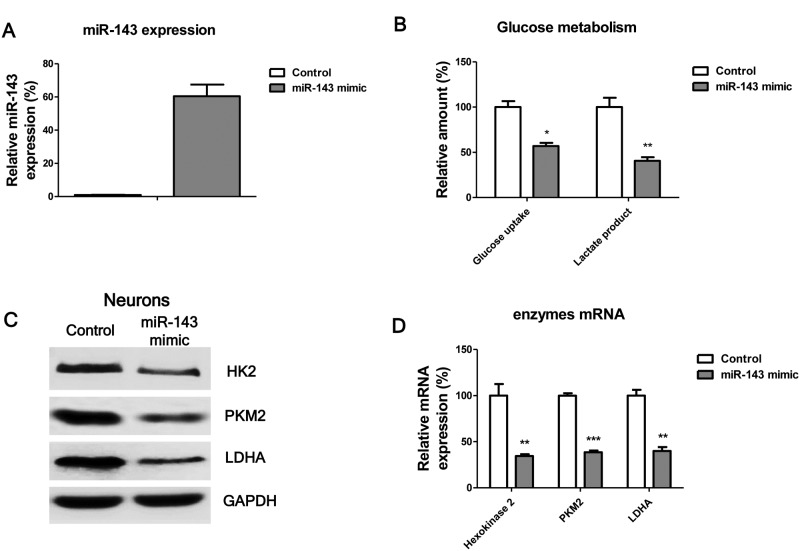
Overexpression of miR-143 suppresses glucose uptake and lactate product of rat neurones (**A**) Expressions of miR-143 were measured in neurones with control mimic transfection or miR-143 mimic transfection. (**B**) Glucose uptake and lactate product were measured in rat neurones without or with overexpression of miR-143. (**C**) The expressions of glycolysis key enzymes were measured by Western blot and (**D**) qRT-PCR in rat neurones without or with overexpression of miR-143. GAPDH was the loading control. Data are representative of three independent experiments. Mean ± S.E.M. *, *P*<0.05; **, *P*<0.01; ***, *P*<0.001.

**Figure 4:**
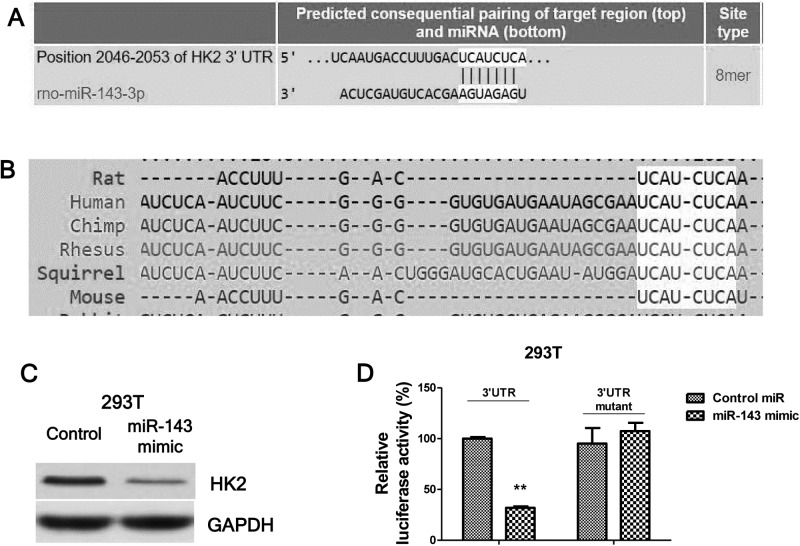
MiR-143 directly targets HK2 in neuron (**A**) Predicted miR-143 binding sites on HK2 3′-UTR region, analyzed from TargetScan. (**B**) The binding sites on HK2 3′-UTR were conserved in multiple species. (**C**) Control mimic or miR-143 mimic were transfected into 293T cells for 48 h, expression of HK2 was examined by Western blot. GAPDH was the loading control. (**D**) Luciferase assay showed the miR-143 could target wild-type 3′-UTR of HK2 but not mutant 3′-UTR of HK2. Data are representative of three independent experiments. Mean ± S.E.M. **, *P*<0.01.

The above results described a correlation amongst miR-143 expression, glucose uptake and lactate product, and IBI in a rat model. Therefore, we hypothesized that miR-143 directly suppresses glucose uptake and lactate product. To test this, we transfected rat neurones with negative control miRNAs or miR-143 mimics for 72 h (Figure 3A). The glucose uptake and lactate production were significantly suppressed by miR-143 (Figure 3B,C). In addition, we observed the glucose metabolism key enzymes were down-regulated by overexpression of miR-143 in rat neurones (Figure 3D,E). To evaluate whether miR-143 could directly target glucose uptake, we searched public miRNAs database, targetscan.org. Through bioinformatics analysis, we found the predicted binding sites by miR-143 in the 3′-UTR of* HK2* mRNA (Figure 4A), indicating that HK2 might be a direct target of miR-143. Furthermore, the predicted binding sites by miR-143 were conserved in multiple species including human and rat (Figure 4B). To verify this prediction, we performed dual-luciferase repoter assays, which contain a wild-type luciferase reporter vector that encoded the 3′-UTR sequence of *HK2* mRNA and a binding site mutant luciferase reporter vector (Figure 4C). As we expected, with the cotransfection of wild-type or mutant luciferase vector and miR-143 mimic together into 293T or rat neuronal cells for 48 h, the luciferase activities in cells were significantly decreased with transfection of vector containing wild-type 3′-UTR segment of HK2 (Figure 4C). However, we did not observe the alterations of luciferase activities in cells with transfection of vector containing binding site mutant 3′-UTR segment of HK2 (Figure 4C). Taken together, our results indicated that miR-143 could directly target HK2 in rat neurones.

### Restoration of HK2 recovers the glucose uptake and lactate product of neuronal cells

**Figure 5:**
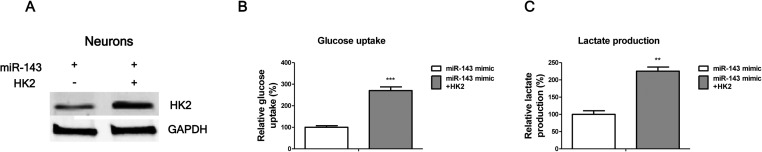
Restoration of HK2 recovers glucose uptake and lactate product of neurones (**A**) Neurones were transfected with miR-143 alone or miR-143 plus HK2 for 48 h, followed by Western blot analysis of the HK2 expression. GAPDH was the loading control. (**B**) Neurones were transfected with miR-143 alone or miR-143 plus HK2 for 48 h, followed by the measurements of glucose uptake, and (**C**) lactate production. Data are representative of three independent experiments. Mean ± S.E.M. **, *P*<0.01; ***, *P*<0.001.

To verify the specificity of the miR-143-mediated HK2 down-regulation, and glucose uptake and lactate product, we performed rescue experiments to restore the HK2 expression in miR-143 overexpressing neurones. Neurones were transiently transfected with control vector, miR-143 alone or miR-143 plus HK2 adenovirus overexpression vector. Results in Figure 5A showed transfection of HK2 in miR-143 overexpressing neurones restoring the original HK2 expression. To assess whether restoration of HK2 could reverse the impaired glucose uptake and lactate product in miR-143 overexpressing neurones, we assayed neurones glucose uptake and lactate production. As we expected, glucose uptake and lactate production were significantly increased by HK2 restoration (Figure 5B,C).

### Inhibition of miR-143 protects the neurones’ death against OGD

**Figure 6:**
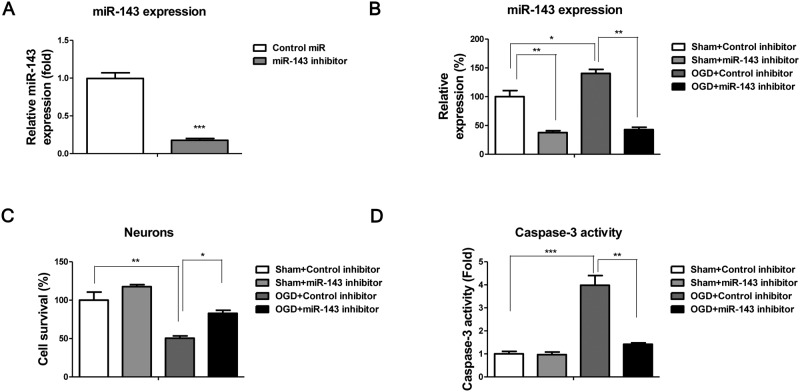
Inhibition of miR-143 protects neurones against IBI (**A**) Neurones were transfected with control or miR-143 inhibitor for 48 h, followed by the detection of miR-143 expressions by qRT-PCR. (**B**) Neurones were transfected with control or miR-143 inhibitor for 48 h, cells were subjected to OGD for 48 h, the expressions of miR-143 were measured by qRT-PCR. (**C**) Neurones were transfected with control or miR-143 inhibitor for 48 h, cells were subjected to OGD for 48 h then the cell viabilities were measured by MTT assay. (**D**) Activities of Caspase-3 were measured in neurones with the indicated treatments. Data are representative of three independent experiments. Mean ± S.E.M. *, *P*<0.05; **, *P*<0.01; ***, *P*<0.001.

The above results demonstrated miR-143 was induced and glucose uptake and lactate product was suppressed subsequently during IBI. The neurones were then transfected with control miRNAs or miR-143 mimic under normal conditions. Results in Figure 6A demonstrated that miR-143 inhibitor could significantly down-regulate miR-143 expressions. Without OGD, we observed overexpression of miR-143 promoted neurones’ death, suggesting inhibition of miR-143 during IBI might protect neurones against cell death. To test this, we pretransfected neurones with control miRNAs or miR-143 inhibitor followed by the OGD treatments. The expression of miR-143 was significantly suppressed by the miR-143 inhibitor under OGD conditions (Figure 6B). Results in Figure 6C illustrated inhibition of miR-143 decreased cell death rate by OGD treatments compared with control inhibitor transfection. In addition, we examined the activity of Caspase-3, which is cell apoptosis marker. Consistently, the activity of Caspase-3 was increased in neurones under OGD treatments but did not change significantly by miR-143 inhibitor transfected neurones (Figure 6D), indicating inhibition of miR-143 during IBI might be a novel therapeutic method for clinical applications.

## Discussion

It is widely studied that miRNAs serve important roles in the development and abnormalities of the brain [14]. Multiple brain-specific miRNAs such as miR-9, miR-183, miR-124a, miR-153, miR-124b, and miR-219 have been reported to be expressed in mouse and human differentiating neurones, suggesting that these miRNAs are essential regulators in mammalian neuronal processes [15].

Increasing evidence revealed that the miRNA-mediated post-transcriptional regulation of target mRNA, plays an important role in the control of neuronal apoptosis and survival under IBI. Previously, miR-29b has been reported to accelerate the neuronal cell death through inhibiting Bcl-2 after IBI [11]. Moreover, during hypoxia, inhibition of miR-210 prevents neuronal cells in hypoxic-IBI in neonatal rats [16], suggesting that miRNAs maybe therapeutic targets against brain injury. In the presentstudy, we investigated the biological roles of miR-143 in IBI using *ex vivo* and *in vivo* rat models. We observed that miR-143 was down-regulated under normal conditions but significantly induced by brain ischemia, which suggests miR-143 plays a pro-apoptotic function during IBI. However, the intricate signaling pathways during cerebral ischemia are not fully understood.

Glucose is the main nutrition used by the brain under normal conditions and more than 95% of the ATP is derived from aerobic glucose oxidation [9]. In synapses, impulse arrives at the presynaptic terminal, leading to the release of neurotransmitter. The neurotransmitter crosses the synapse to bind with receptors on the membrane of the postsynaptic cell, with the opening of ion-specific channels in the postsynaptic membrane [17]. These processes are energy-consuming that require a constant supply of glucose and oxygen to the neurones. Therefore, understanding the regulations of glucose metabolism during normal or ischemia are import for the development of therapeutic agents against brain ischemia. Recent studies illustrated stimulating glucose metabolism may be an effective therapeutic approach to decrease the severity of ischemic injury [18,19]. However, the regulation of glucose metabolism during brain ischemia remains poorly understood. Our results demonstrated an impaired glucose metabolism profiling during IBI in rat *in vivo* and *ex vivo* models, suggesting a negative correlation between glucose metabolism and brain ischemia. Moreover, we observed overexpression of miR-143 suppressed glucose metabolism through directly targetting HK2, illustrating an ischemia-miR-143-HK2 axis. Importantly, inhibition of miR-143 in rat neurones protected neuronal cells against the OGD-induced cell death. Interestingly, it has been demonstrated that moderate increase in lactate post-ischemia has neuroprotective effect [20], supporting our study that recovery of glucose metabolism could protect neurones. The detailed mechanisms are still under investigation. In summary, we report that miR-143 is induced during IBI, leading to the suppression of glucose metabolism through direct targetting HK2. Inhibition of miR-143 during IBI demonstrated a neuroprotective effect, providing a new aspect on the development of therapeutic approaches for brain ischemia treatments.

## Abbreviations

HK2: hexokinase 2
IBI: ischemic brain injury
MCAO: middle cerebral artery occlusion
OGD: oxygen-glucose deprivation

## References

[1] Jivad N. and Rabiei Z. (2015) Review on herbal medicine on brain ischemia and reperfusion. Asian Pac. J. Trop. Biomed. 5, 789–795

[2] Hinkle J.L. and Guanci M.M. (2007) Acute ischemic stroke review. J. Neurosci. Nurs. 39, 285–293 17966295 10.1097/01376517-200710000-00005

[3] Pan J., Konstas A.-A., Bateman B., Ortolano G.A. and Pile-Spellman J. (2007) Reperfusion injury following cerebral ischemia: pathophysiology, MR imaging, and potential therapies. Neuroradiology 49, 93–102 17177065 10.1007/s00234-006-0183-z PMC1786189

[4] Xia Z., Chen Y., Fan Q. and Xue M. (2014) Oxidative stress-mediated reperfusion injury: mechanism and therapies. Oxid. Med. Cell. Longev. 2014, 373081 24803980 10.1155/2014/373081 PMC3997101

[5] Jonas S. and Izaurralde E. (2015) Towards a molecular understanding of microRNA-mediated gene silencing. Nat. Rev. Genet. 16, 421–433 26077373 10.1038/nrg3965

[6] Min X.L., Wang T.Y., Cao Y., Liu J., Li J.T. and Wang T.H. (2015) MicroRNAs: a novel promising therapeutic target for cerebral ischemia/reperfusion injury? Neural Regen. Res. 10, 1799–1808 26807114 10.4103/1673-5374.170302 PMC4705791

[7] Zhai F., Zhang X., Guan Y., Yang X., Li Y., Song G. (2012) Expression profiles of microRNAs after focal cerebral ischemia/reperfusion injury in rats. Neural Regen. Res 7, 917–923 25722676 10.3969/j.issn.1673-5374.2012.12.007 PMC4341287

[8] Jiang Y., Li L., Tan X., Liu B., Zhang Y. and Li C. (2015) *miR-210* mediates vagus nerve stimulation-induced antioxidant stress and anti-apoptosis reactions following cerebral ischemia/reperfusion injury in rats. J. Neurochem. 134, 173–181 25783636 10.1111/jnc.13097

[9] Mergenthaler P., Lindauer U., Dienel G.A. and Meisel A. (2013) Sugar for the brain: the role of glucose in physiological and pathological brain function. Trends Neurosci. 36, 587–597 23968694 10.1016/j.tins.2013.07.001 PMC3900881

[10] Ramamoorthy P. and Shi H. (2014) Ischemia induces different levels of hypoxia inducible factor-1α protein expression in interneurons and pyramidal neurons. Acta Neuropathol. Commun. 2, 51 24887017 10.1186/2051-5960-2-51 PMC4035094

[11] Shi G., Liu Y., Liu T., Yan W., Liu X., Wang Y. (2012) Upregulated *miR-29b* promotes neuronal cell death by inhibiting Bcl2L2 after ischemic brain injury. Exp. Brain Res. 216, 225–230 22094713 10.1007/s00221-011-2925-3

[12] Panasyuk G., Espeillac C., Chauvin C., Pradelli L.A., Horie Y., Suzuki A. (2012) PPARγ contributes to PKM2 and HK2 expression in fatty liver. Nat. Commun. 3, 672 22334075 10.1038/ncomms1667 PMC3293420

[13] Lee J.M., Grabb M.C., Zipfel G.J. and Choi D.W. (2000) Brain tissue responses to ischemia. J. Clin. Invest. 106, 723–731 10995780 10.1172/JCI11003 PMC381398

[14] Petri R., Malmevik J., Fasching L., Åkerblom M. and Jakobsson J. (2014) miRNAs in brain development. Exp. Cell Res. 321, 84–89 24099990 10.1016/j.yexcr.2013.09.022

[15] Follert P., Cremer H. and Béclin C. (2014) MicroRNAs in brain development and function: a matter of flexibility and stability. Front. Mol. Neurosci. 7, 5 24570654 10.3389/fnmol.2014.00005 PMC3916726

[16] Ma Q., Dasgupta C., Li Y., Bajwa N.M., Xiong F., Harding B. (2016) Inhibition of microRNA-210 provides neuroprotection in hypoxic-ischemic brain injury in neonatal rats. Neurobiol. Dis. 89, 202–212 26875527 10.1016/j.nbd.2016.02.011 PMC4785034

[17] Kavalali E.T. (2015) The mechanisms and functions of spontaneous neurotransmitter release. Nat. Rev. Neurosci. 16, 5–16 25524119 10.1038/nrn3875

[18] Jalloh I., Carpenter K.L., Helmy A., Carpenter T.A., Menon D.K. and Hutchinson P.J. (2015) Glucose metabolism following human traumatic brain injury: methods of assessment and pathophysiological findings. Metab. Brain Dis. 30, 615–632 25413449 10.1007/s11011-014-9628-y PMC4555200

[19] Brooks G.A. and Martin N.A. (2015) Cerebral metabolism following traumatic brain injury: new discoveries with implications for treatment. Front. Neurosci. 8, 408 25709562 10.3389/fnins.2014.00408 PMC4321351

[20] Horn T. and Klein J. (2013) Neuroprotective effects of lactate in brain ischemia: dependence on anesthetic drugs. Neurochem. Int. 62, 251–257 23298645 10.1016/j.neuint.2012.12.017

